# Usefulness of ELISA Using Total Antibody against Plant-Expressed Recombinant Nucleocapsid Protein of SARS-CoV-2

**DOI:** 10.1128/Spectrum.00672-21

**Published:** 2021-11-24

**Authors:** Misbah Tariq, Jian Hur, Jun-Won Seo, Da Young Kim, Na Ra Yun, You Mi Lee, Mi-Seon Bang, Seong Yeon Hwang, Choon-Mee Kim, Ju-Hyung Lee, Kyoung-Ho Song, Hyunju Lee, Jongtak Jung, Ji Young Park, Hong Bin Kim, Eu Suk Kim, Sangmin Lee, Dong-Min Kim

**Affiliations:** a Department of Internal Medicine, College of Medicine, Chosun University grid.254187.dgrid.464555.3grid.254187.dgrid.464555.3 , Gwangju, Republic of Korea; b Premedical Science, College of Medicine, Chosun University grid.254187.dgrid.464555.3grid.254187.dgrid.464555.3 , Gwangju, Republic of Korea; c Department of Infectious Disease Internal Medicine, College of Medicine, Yeungnam University, Gyeongsan, Republic of Korea; d Department of Preventive Medicine, Jeonbuk National University Medical School, Jeonju, Republic of Korea; e Department of Internal Medicine, Seoul National University Bundang Hospital, College of Medicine, Seongnam, Republic of Korea; f Department of Pediatrics, Seoul National University Bundang Hospital, Seongnam, Republic of Korea; g BioApplications Inc., Pohang, Republic of Korea; Keck School of Medicine of the University of Southern California

**Keywords:** SARS-CoV-2, COVID-19, ELISA, total antibody, nucleoprotein, *E. coli*, plant

## Abstract

Here, we aimed to investigate the diagnostic value of a serological assay using the nucleocapsid protein developed for severe acute respiratory syndrome coronavirus 2 (SARS-CoV-2) detection and evaluated its performance using three commercial enzyme-linked immunosorbent assays (ELISAs), namely, Standard E 2019 novel coronavirus disease (COVID-19) total antibody (Ab) ELISA (SD Biosensor), and EDI novel coronavirus COVID-19 IgG and IgM ELISA. A recombinant nucleocapsid protein (rNP) was expressed from plants and Escherichia coli for the detection of serum total Ab. We prospectively collected 141 serum samples from 32 patients with reverse transcription-PCR (RT-PCR)-confirmed COVID-19 and determined the sensitivity and dynamics of their total Ab response. Specificity was evaluated using 158 prepandemic samples. To validate the assays, we evaluated the performance using two different cutoff values. The sensitivity and specificity for each assay were as follows: 92.91% and 94.30% (plant-rNP), 83.69% and 98.73% (SD Biosensor), 75.89% and 98.10% (E. coli-rNP), 76.47% and 100% (EDI-IgG), and 80.39% and 80% (EDI-IgM). The plant-based rNP showed the highest sensitivity and area under the receiver operating characteristic (ROC) curve (0.980) among all the assays (*P* < 0.05). The seroconversion rate for total Ab increased sequentially with disease progression, with a sensitivity of 100% after 10 to 12 days of post-symptom onset (PSO) for both rNP-plant-based and SD Biosensor ELISAs. After 2 weeks of PSO, the seroconversion rates were >80% and 100% for EDI-IgM and EDI-IgG ELISA, respectively. Seroconversion occurred earlier with rNP plant-based ELISA (5 days PSO) compared with E. coli*-*based (7 days PSO) and SD Biosensor (8 days PSO) ELISA. We determined that rNP produced in plants enables the robust detection of SARS-CoV-2 total Abs. The assay can be used for serosurvey and complementary diagnosis of COVID-19.

**IMPORTANCE** At present, the principal diagnostic methods for COVID-19 comprise the identification of viral nucleic acid by genetic approaches, including PCR-based techniques or next-generation sequencing. However, there is an urgent need for validated serological assays which are crucial for the understanding of immune responses against SARS-CoV-2. In this study, a highly sensitive and specific serological antibody assay was developed for the detection of SARS-CoV-2 with an overall accuracy of 93.56% using a recombinant nucleoprotein expressed from plants.

## INTRODUCTION

In December 2019, a novel coronavirus named severe acute respiratory syndrome coronavirus 2 (SARS-CoV-2) was first recognized in Wuhan City, Hubei Province, China, and has since spread rapidly and caused the 2019 novel coronavirus disease (COVID-19) pandemic.

At present, the principal diagnostic methods of COVID-19 include the identification of viral nucleic acids by genetic approaches, including PCR-based techniques and next-generation sequencing (1). However, there is an urgent need for validated serological assays which are well known to assist in many extremely relevant applications. First, serological assays are crucial for the understanding of immune responses against SARS-CoV-2, both quantitatively and qualitatively. Second, serosurveys are essential for ascertaining the specific rate of infection, which is a key parameter to precisely determine its fatality rate. Third, these assays can contribute to the development of convalescent plasma therapy by means of recognizing individuals who have mounted substantial antibody responses and may act as plasma donors. Finally, serological assays can help determine the antibody responses that complement the protection from SARS-CoV-2 (2).

The coronavirus has four main structural proteins, namely, membrane (M), envelope (E), spike (S), and nucleocapsid (N) (3), with S and N being the main immunogens (4). The S protein is found on the surface of viral particles and consists of two major functional subunits responsible for viral cell membrane binding (S1) and fusion (S2) to host cell receptors (4). The N protein is one of the most important structural proteins involved in the replication and transmission of viral RNA and interferes with the host cell cycle (5). Furthermore, in many coronaviruses, the N protein is highly immunogenic and abundantly expressed during the course of infection (6). Consequently, it may act as a potential antigen for the serological diagnosis of COVID-19, as with many developed diagnostic immunoassays for SARS-CoV-2 (6–8).

The validity of a serological assay depends on its sensitivity and specificity. Here, we report the development of a serological assay for the detection of total antibody (Ab), and its performance against plant- and Escherichia coli-expressed recombinant nucleoproteins (rNPs) of SARS-CoV-2 was evaluated using enzyme-linked immunosorbent assays (ELISAs). The study was conducted on serial serum samples obtained from patients infected with SARS-CoV-2. To date, very few studies have assessed the accuracy of a serological assay in terms of sensitivity and specificity (9–11). To the best of our knowledge, the present study is the first to compare the diagnostic accuracy of nucleoproteins expressed from both plants and E. coli for the detection of SARS-CoV-2. We further investigated the analytical validity of total Ab ELISA in the identification of seroconversion and compared its performance with that of a commercially available Standard E COVID-19 total Ab ELISA (SD Biosensor) and EDI novel coronavirus COVID-19 IgG and IgM ELISAs.

## RESULTS

### Definition of patients infected with SARS-CoV-2.

A positive case was defined as a person with confirmed COVID-19 in compliance with diagnostic measures involving viral isolation and/or real-time reverse transcription-PCR (rRT-PCR) targeting two genes. Negative sera were obtained before the COVID-19 pandemic. These samples were used to characterize the true positives and true negatives of the ELISAs, by evaluating their conformity with the actual SARS-CoV-2 diagnosis on the basis of two cutoff values.

### SARS-CoV-2 antigen expression in plant and E. coli.

The purity of plant- and E. coli*-*based rNPs was assessed by SDS-PAGE and Coomassie blue staining (see Fig. S1A and C in the supplemental material). The antigenicity of the recombinant proteins was evaluated by Western blotting using patient serum samples (Fig. S1B and D). The results showed the expected molecular weights of ∼48 kDa and ∼46 kDa for plant- and E. coli*-*based rNPs, respectively.

### Determination of optimal cutoff value for ELISAs.

Two different approaches were used to calculate the cutoff values, and the optimal value was selected based on the higher accuracy obtained (Table 1). The recommended cutoff values by using the receiver operating characteristic (ROC) curve were 0.5, 1.8, 0.18, 0.28, and 0.09 for the plant rNP-total Ab-, E. coli rNP-total Ab-, SD Biosensor-total Ab-, EDI-IgG-, and EDI-IgM-based ELISAs, respectively. The acquired cutoff values by using mean + 3 SD were 0.7 and 2.2 for the plant rNP- and E. coli rNP-based ELISAs, respectively. For SD-Biosensor-total Ab, the obtained cutoff value was 0.36. For EDI-IgG- and IgM-based ELISAs, the acquired ranges of negative and positive cutoff values were 0.32 to 0.39 and 0.18 to 0.22, respectively (Table 1).

**TABLE 1:** Performance of in-house rNP plant- and E. coli-based, SD Biosensor total Ab, and EDI novel coronavirus COVID-19 IgG and IgM ELISAs^*a*^

Method		Data by ELISA				
		rNP (plant based)	rNP (E. coli based)	SD Biosensor total Ab	EDI-IgG	EDI-IgM
ROC^*b*^ curve	Cutoff	0.5	1.8	0.18	0.28	0.09
	True positive (*n*)	131/141	107/141	118/141	39/51	41/51
	True negative (*n*)	149/158	155/158	156/158	20/20	16/20
	Intermediate (*n*)	N/A^*d*^	N/A	N/A	N/A	N/A
	Sensitivity (% [95% CI^*c*^])	92.91 (87.34–96.55)^*e*^	75.89 (67.97–82.69)	83.69 (76.54–89.37)	76.47 (62.51–87.21)	80.39 (66.88–90.18)
	Specificity (% [95% CI])	94.30 (89.46–97.36)	98.10 (94.55–99.61)	98.73 (95.50–99.85)	100 (83.16–100)	80 (56.34–94.27)
	Accuracy (% [95% CI])	93.65 (90.25–96.13)	87.63 (83.35–91.14)	91.64 (87.90–94.52)	83.10 (72.34–90.95)	80.28 (69.14–88.78)
	AUC	0.980 (0.957–0.993)^*e*^	0.929 (0.893–0.955)	0.923 (0.887–0.951)	0.894 (0.798–0.955)	0.805 (0.694–0.890)
Mean + 3 SD or range/value	Cutoff	0.7	2.2	0.36	0.32–0.39	0.18–0.22
	True positive (*n*)	118/141	90/141	115/141	37/51	24/51
	True negative (*n*)	158/158	158/158	157/158	20/20	19/20
	Intermediate (*n*)	N/A	N/A	N/A	2/71	1/71
	Sensitivity (% [95% CI])	83.69 (76.54–89.37)^*f*^	63.83 (55.32–71.75)	81.56 (74.16–87.59)	72.55 (58.26–84.11)^*g*^	45.28 (31.56–59.55)
	Specificity (% [95% CI])	100 (97.69–100)	100 (97.69–100)	99.37 (96.52–99.98)	100 (83.16–100)	95 (75.13–99.87)
	Accuracy (% [95% CI])	92.31 (88.68–95.06)	82.94 (78.19–87.03)	90.97 (87.13–93.96)	80.28 (69.14–88.78)	58.90 (46.77–70.29)

a The data set incorporates all 299 sample results for rNP plant-based, E. coli*-*based, and SD Biosensor-Total Ab ELISA, obtained from 32 (symptomatic and asymptomatic) patients with COVID-19 (141 samples) and 158 samples from negative controls, while 71 samples were included for EDI novel coronavirus COVID-19 IgG and IgM ELISA containing 51 samples from 21 patients (symptomatic and asymptomatic) with COVID-19 and 20 samples from negative controls.

b ROC, receiver operating characteristic.

c 95% CI, 95% confidence interval.

d N/A, not applicable.

e *P* < 0.05 vs all.

f *P* < 0.05 vs all except SD Biosensor total Ab.

g *P* < 0.05 vs versus EDI-IgM.

Both in-house and commercial assay cutoff values acquired from the ROC curve provided higher accuracies; therefore, these values were selected for further analysis.

### Specificity of the ELISAs.

To verify the specificity (95% confidence interval) of the assays, 158 pre-COVID-19 serum samples were analyzed. The specificity varied between 94.30% (89.46% to 97.36%) and 98.10% (94.55% to 99.61%) for the plant rNP- and E. coli rNP-based assays, respectively (*P *= 0.109). Two samples were false positive for SD Biosensor with a specificity of 98.73% (95.50% to 99.85%).

With the use of 20 pre-COVID-19 sera, the specificity was 100% (83.16% to 100%) and 80% (56.34% to 94.27%) for EDI-IgG and IgM ELISA, respectively (*P* = 0.125) (Table 1).

### Sensitivity and comparative analysis for overall diagnostic performance.

**FIG 1:**
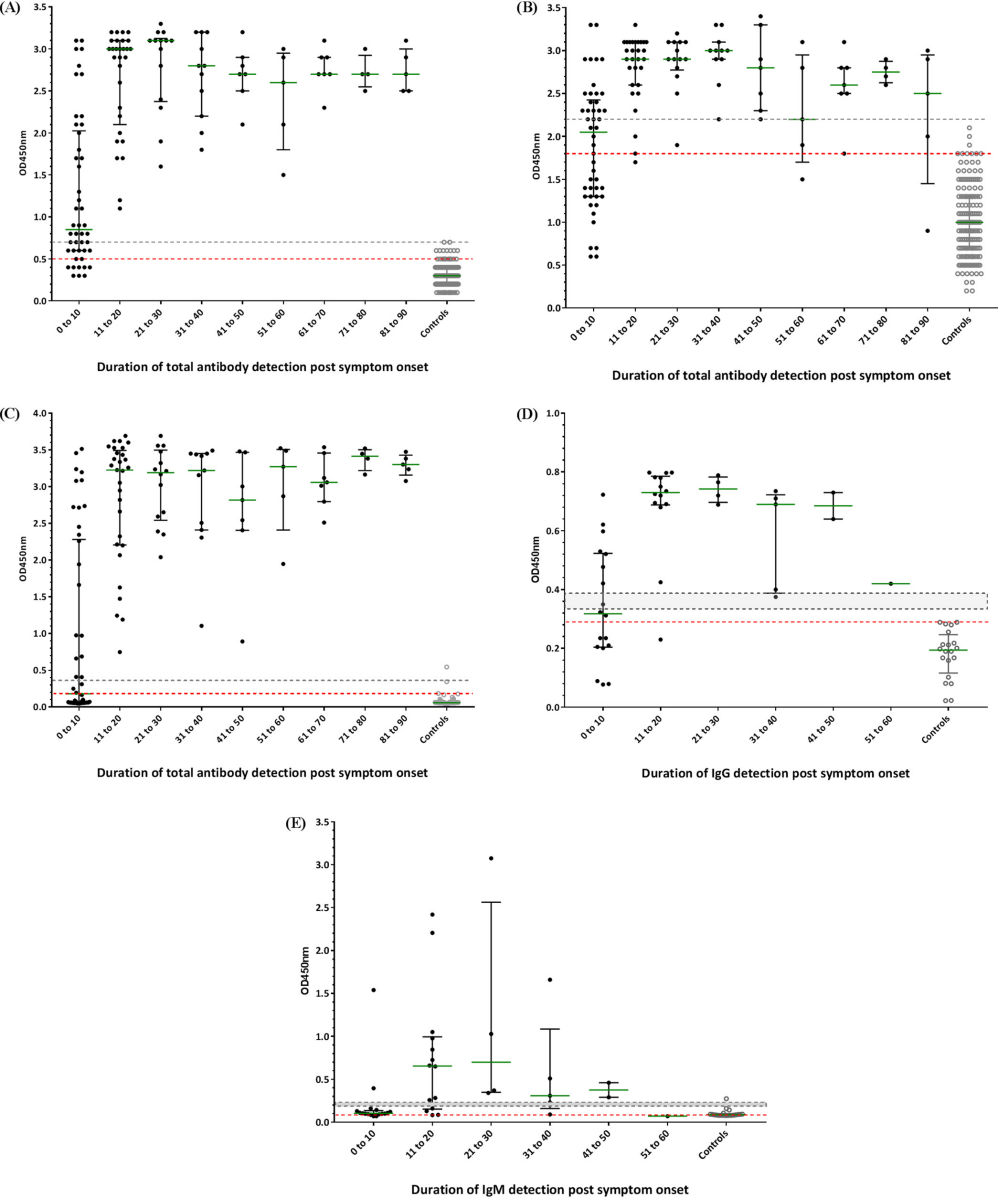
Optical density at 450 nm (OD_450_) for antibody detection by days after symptom onset. Plant-based rNP (A), E. coli-based rNP (B), SD Biosensor total Ab (C), EDI-IgG (D), and EDI-IgM (E) ELISAs. The gray line shows the calculated cutoff values by mean + 3 SD or range recommended by commercial assay. The red line shows the cutoff values recommended by the ROC curve. Green lines indicate the median with interquartile ranges. The data set contained 286 samples for rNP plant-based, rNP E. coli-based, and SD Biosensor total Ab ELISAs, including 128 samples from 30 symptomatic COVID-19 patients and 158 samples from negative controls. For the EDI-IgG and -IgM ELISAs, 64 samples were analyzed, including 44 samples from 19 symptomatic COVID-19 patients and 20 samples from negative controls. mean + 3 SD, mean optical density plus 3-fold of standard deviation. ROC, receiver operating characteristic.

The overall sensitivity for 141 serum specimens varied between 92.91% (87.34% to 96.55%) for plant rNP, 83.69% (76.54% to 89.37%) for SD Biosensor, and 75.89% (67.97% to 82.69%) for E. coli rNP (Table 1). All false negatives of the plant rNP-based and SD Biosensor ELISA were obtained from samples acquired within 10 days post-symptom onset (PSO) (Fig. 1A and C).

The sensitivity of the EDI-ELISA was calculated using 51 samples. The overall sensitivity of EDI-IgM ELISA was 80.39% (66.88% to 90.18%), which was slightly greater than that observed for EDI-IgG ELISA at 76.47% (62.51% to 87.21%) (*P* = 0.8145). Among all the assays, the plant rNP-based ELISA showed the highest sensitivity (92.91%) and accuracy (93.65%; 90.25% to 96.13%), with a statistically significant difference (*P* < 0.05).

### Dynamic trend to seropositivity against SARS-CoV-2 relative to the duration of illness.

The samples were grouped by week to observe the progression of antibodies. For the rNP-based and SD Biosensor ELISA, 128 tested samples were obtained from 30 symptomatic patients. Of these samples, 28 were collected between the day of symptom onset (day 0) and 6 days PSO (week 1), 25 between 7 and 13 days PSO (week 2), 21 between 14 and 20 days PSO (week 3), and 54 after 20 days PSO (>3 weeks). For the EDI-ELISA, 44 samples were obtained from 19 symptomatic patients. Of these samples, 11 were analyzed for week 1, 10 for week 2, 11 for week 3, and 12 for >3 weeks (Table 2; see Fig. S2 in the supplemental material).

**TABLE 2:** Sensitivity of in-house rNP plant-based, E. coli-based, SD Biosensor total Ab, and EDI novel coronavirus COVID-19 IgG and IgM ELISAs with the duration of illness^*a*^

Method by wk^*b*^	Sensitivity (% [*n*, 95%CI^*c*^]) by assay				
	rNP (plant based)	rNP (E. coli based)	SD Biosensor total Ab	EDI-IgG^*d*^	EDI-IgM^*d*^
ROC curve^*e*^					
1	67.86 (19/28, 47.65–84.12)	50.00 (14/28, 30.65–69.35)	67.86 (19/28, 47.65–84.12)	36.36 (4/11, 10.93–69.21)	72.73 (8/11, 39.03–93.98)
2	96.15 (25/26, 80.36–99.90)	69.23 (18/26, 48.21–85.67)	84.62 (22/26, 65.13–95.64)	80.00 (8/10, 44.39–97.48)	80.00 (8/10, 44.39–97.48)
3	100 (21/21, 83.89–100)	95.24 (20/21, 76.18–99.88)	100 (21/21, 83.89–100)	100 (11/11, 71.51–100)	90.91 (10/11, 58.72–99.77)
>3	100 (53/53, 93.40–100)	94.34 (50/53, 84.34–98.82)	100 (53/53, 93.40–100)	100 (12/12, 73.54–100)	83.33 (10/12, 51.59–97.91)
Mean + 3 SD or range/value^*f*^					
1	50.00 (14/28, 30.65–69.35)	35.71 (10/28, 18.64–55.93)	32.14 (9/28, 15.88–52.35)	27.27 (3/11, 6.02–60.97)	18.18 (2/11, 2.28–51.78)
2	88.77 (21/26, 60.65–93.45)	53.85 (14/26, 33.37–73.41)	73.08 (19/26, 52.21–88.43)	70.00 (7/10, 34.75–93.33)	20.00 (2/10, 2.52–55.61)
3	100 (21/21, 83.89–100)	95.24 (20/21, 76.18–99.88)	100 (21/21, 83.89–100)	100 (11/11, 71.51–100)	81.82 (9/11, 48.22–97.72)
>3	100 (53/53, 93.28–100)	83.02 (44/53, 70.20–91.93)	100 (53/53, 93.28–100)	100 (12/12, 73.54–100)	83.33 (10/12, 51.59–97.91)

a The data set contained 128 samples for rNP plant-based, rNP E. coli-based, and SD Biosensor total Ab assays from 30 symptomatic COVID-19 patients.

b Wk 1, 0–6 days PSO; wk 2, 7–13 days PSO; wk 3, 14–20 days PSO; >wk 3, ≥21 to 91 days PSO.

c 95% CI, 95% confidence interval.

d For the EDI-IgG and -IgM ELISA assays, 44 samples were analyzed from 19 symptomatic COVID-19 patients.

e ROC, receiver operating characteristic.

f Mean + 3 SD, mean optical density plus 3-fold standard deviation.

**FIG 2:**
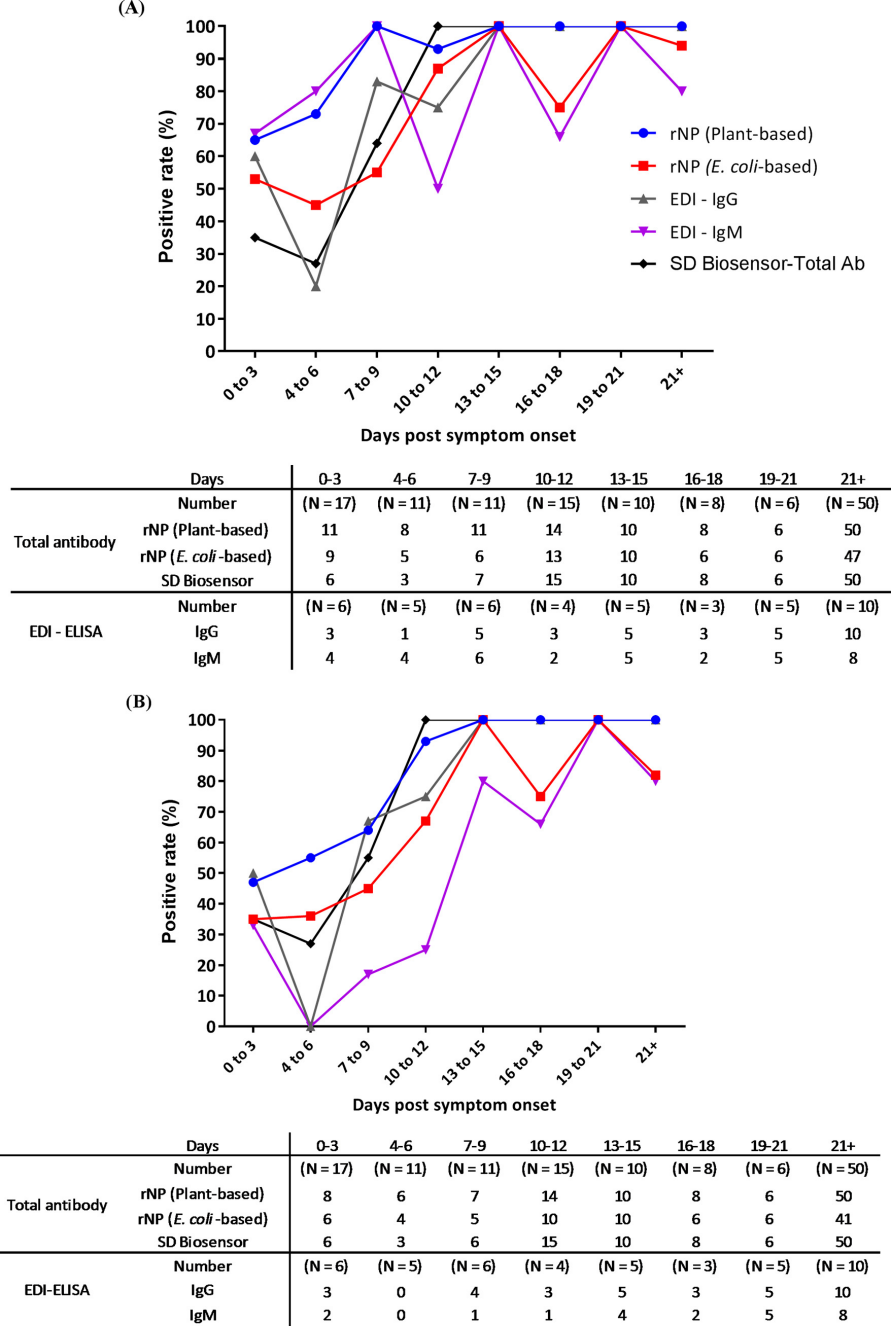
Graph of the positive rate of SARS-CoV-2-specific total antibodies, IgG, and IgM by days post-symptom onset. (A) Positive rate obtained from recommended cutoff values by ROC curve. (B) Positive rate obtained from calculated cutoff values by mean + 3 SD or range recommended by commercial assay. The data set contains 128 samples for rNP plant-based and rNP E. coli-based assays from 30 symptomatic COVID-19 patients. For EDI-IgG ELISA, 44 samples were analyzed from 19 symptomatic COVID-19 patients. The cutoffs recommended by ROC curve were 0.5, 1.8, 0.18, 0.28, and 0.09 for rNP plant-based, rNP E. coli-based, SD Biosensor total Ab, EDI-IgG, and EDI-IgM ELISAs, respectively. The calculated cutoff values by mean + 3 SD or range/value recommended by commercial assay were 0.7, 2.2, 0.36, 0.32 to 0.39, and 0.18 to 0.22 for rNP plant-based, rNP E. coli- based, SD Biosensor test Ab, EDI-IgG, and EDI-IgM ELISAs, respectively. The borderline results obtained from the EDI commercial assay were considered positive.

The sensitivity of EDI-IgM was highest in week 1 at 72.73% (39.03% to 93.98%) followed by plant rNPs at 67.86% (47.65% to 84.12%). The sensitivity of all assays increased during week 2. For plant rNPs, SD Biosensor, and EDI-IgG ELISA, the seroconverted samples reached a plateau at 100% at 10 and 12 days PSO (Fig. 1A, C, and D and Fig. 2). None of the patients became seronegative after the first positive result obtained with plant rNP, SD Biosensor, and EDI-IgG assays; however, no such pattern was observed in the samples tested with E. coli rNP and EDI-IgM ELISAs (Fig. 2).

The seroconversion of total Ab was observed to occur as early as the day of symptom onset, with a median of 5 days PSO (interquartile range [IQR], 1 to 9 days) for plant rNP, 7 days PSO (IQR, 1 to 10 days) for E. coli rNP, and 8 days PSO (IQR, 5 to 10 days) for SD Biosensor ELISA. For EDI-IgG and-IgM ELISAs, the day of seropositivity could not be identified, as complete serial samples were not analyzed using these kits.

### ROC analysis.

**FIG 3:**
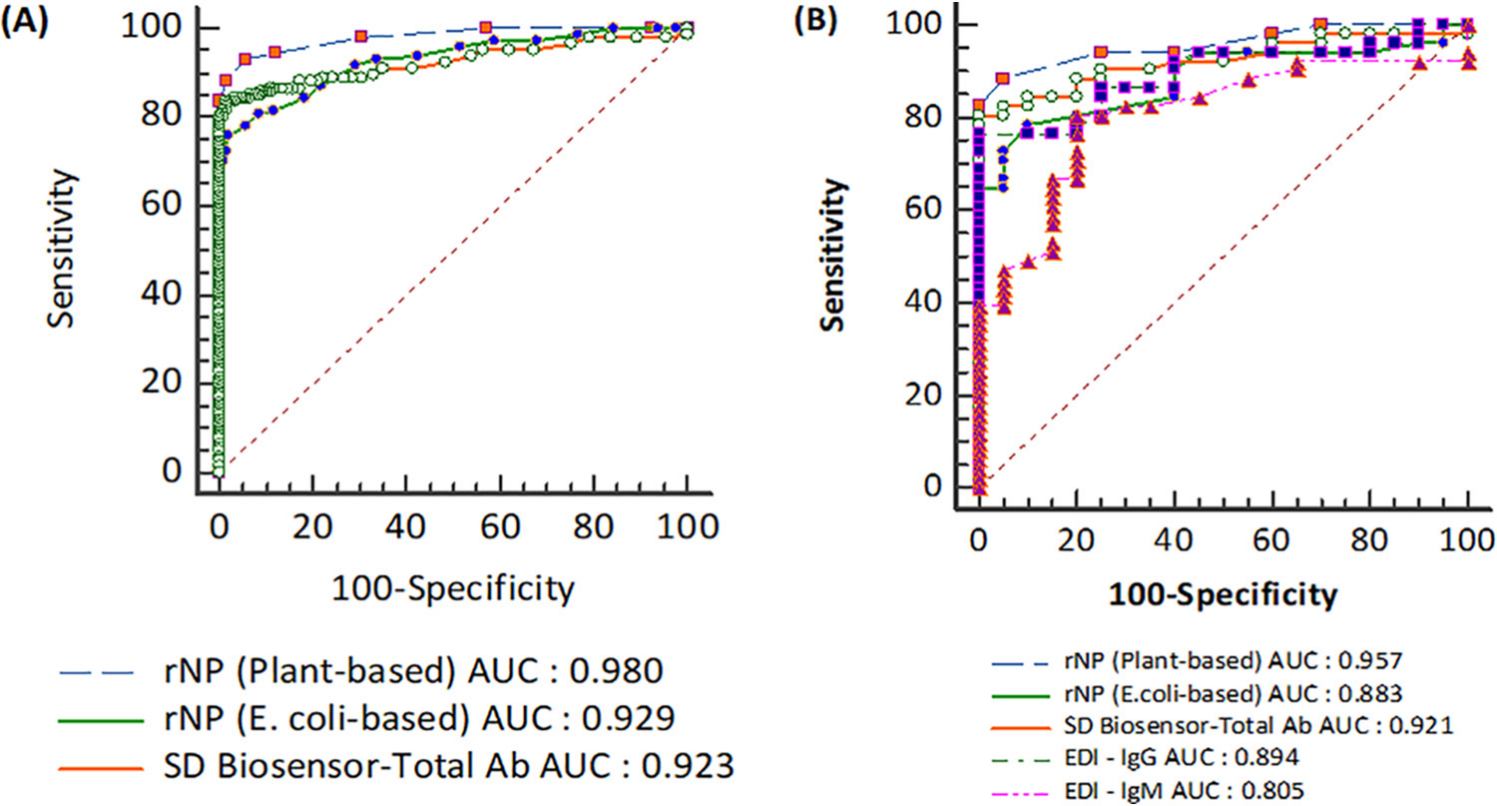
Receiver operating characteristic (ROC) curves for the evaluation and comparison of diagnostic accuracy. (A) Plant rNP-based, E. coli rNP-based, and SD Biosensor total Ab ELISAs. (B) Plant rNP-based, E. coli rNP-based, SD Biosensor total Ab, EDI-IgG, and EDI-IgM ELISAs. The data set contain all 299 samples for rNP plant-based, rNP E. coli-based, and SD Biosensor total Ab ELISAs, including 141 samples from 32 patients with COVID-19 and 158 samples from negative controls. For comparison with EDI-IgG and -IgM ELISA, the data set contains 71 samples, including 51 samples from 21 patients with COVID-19 and 20 samples from negative controls.

The area under the concentration-time curve (AUC) obtained from the ROC analysis provides a good parameter for the diagnostic power of a specific test and was compared among the different ELISAs (Fig. 3). Compared with all assays, the plant-based rNPs had the significantly highest measure at 0.957 (0.881 to 0.991; *P* < 0.05).

### Correlation analysis between ELISAs.

All tests were significantly correlated with each other (*P* < 0.0001), as assessed by Pearson’s correlation coefficient (*r*). The strongest positive correlation was observed in plant-based rNP and EDI-IgG ELISAs, with an *r* of 0.9077, followed by plant-based rNP and SD Biosensor (*r* = 0.887) and E. coli-based rNP (*r* = 0.869). All assays showed a moderate positive correlation (*r* = 0.5117 to 0.5560) with EDI-IgM ELISA (Table 3).

**TABLE 3:** Correlation analysis of the OD values between in-house rNP plant-based, E. coli-based, SD Biosensor total Ab, and EDI novel coronavirus COVID-19 IgG and IgM ELISAs

ELISA	Parameter	Data by ELISA				
		rNP (plant based)	rNP (E. coli based)	SD Biosensor total Ab	EDI-IgG	EDI-IgM
rNP (plant based)	Correlation coefficient		0.869	0.887	0.908	0.523
	*P* value^*a*^		<0.0001	<0.0001	<0.0001	<0.0001
	*n*		299	299	71	71
rNP (E. coli based)	Correlation coefficient^*b*^	0.869		0.782	0.821	0.556
	*P* value	<0.0001		<0.0001	<0.0001	<0.0001
	*n* ^*c*^	299		299	71	71
SD Biosensor total Ab	Correlation coefficient	0.887	0.782		0.745	0.531
	*P* value	<0.0001	<0.0001		<0.0001	<0.0001
	*n*	299	299		71	71
EDI-IgG	Correlation coefficient	0.908	0.821	0.745		0.512
	*P* value	<0.0001	<0.0001	<0.0001		<0.0001
	*n*	71	71	71		71
EDI-IgM	Correlation coefficient	0.523	0.523	0.531	0.512	
	*P* value	<0.0001	<0.0001	<0.0001	<0.0001	
	*n*	71	71	71	71	

a The analysis was performed with Pearson's correlation coefficient.

b The interpretations of correlation coefficient (*r*) were characterized as follows: 0.00–0.19, very weak; 0.20–0.39, weak; 0.40–0.59, moderate; 0.60–0.799, strong; and 0.80–1.00, very strong.

c *n*, no. of samples.

### Identification of asymptomatic infection via contact tracing.

In our study, two patients (2/32, 6.25%) had occult asymptomatic infections and were identified through contact tracing. Throughout the follow-up period, both patients remained asymptomatic. The plant-based rNP and SD Biosensor assays detected seroconversion on the day of hospital admission, but the E. coli-rNP assay detected seropositivity on the 10th day of hospitalization. The antibody response remained positive during the follow-up period of 3 weeks. These patients were also found to be positive by both EDI-ELISAs; however, the day of seropositivity could not be identified, as a complete set of samples was not analyzed by these assays.

## DISCUSSION

Validated serological assays for SARS-CoV-2 are currently insufficient and are nevertheless promptly required for efficient diagnosis, contact tracing, epidemiologic clarification, and the advancement of vaccine studies. Previous data on the SARS-CoV-2 epidemic showed that evaluation of the antibody response was effective for serodiagnosis (12, 13). In the present study, we developed and evaluated ELISA to detect antinucleocapsid antibodies (total Ab) using an rNP antigen expressed in plants and E. coli and compared their performance with three commercial ELISAs (SD Biosensor Standard E COVID-19 total Ab ELISA and EDI novel coronavirus COVID-19 IgG and IgM).

Nucleoproteins can be easily expressed and purified in vast quantities in prokaryotic and eukaryotic hosts. Plant-based expression systems offer some advantages over more widely held insect or mammalian systems since the required media or growth conditions are optimized and inexpensive (14). The advantages over bacterial or yeast systems are that posttranslational modifications are somewhat similar to mammalian cell lines, and they lack contaminating pathogens or endotoxins that can cause problems with the purification of the desired protein (14, 15). The deficiency of precise protein glycosylation and yield of recombinant proteins are considered disadvantages for using plant-expressed proteins. However, more recently, Nicotiana benthamiana has been increasingly accepted as the preferred protein expression host because of its compliance with higher levels of transient gene expression, rapid generation of biomass, a defective posttranscriptional gene silencing system, and engineering strategies in the secretory pathway of plants, of which all can overcome the difficulty of low yield (16, 17).

Our results of the total Ab response against SARS-CoV-2 indicated the analytical validation of the serological test for COVID-19 diagnosis and showed superior performance with plant-expressed recombinant proteins. Moreover, the results of the plant-expressed protein showed excellent correlation with the widely used commercial IgG (EDI) and total Ab (SD Biosensor) ELISA kits. Remarkably, all patients seroconverted in the early 2 weeks, thus demonstrating 100% seropositivity of total Ab by 10 days PSO; these findings corroborated with those of recently published reports (9, 11, 18). Additionally, our results indicated that the total Ab persisted well beyond 2 months of PSO in the 17 serum samples obtained from 13 symptomatic patients.

Using our ELISA with plant-expressed rNP, we demonstrated excellent sensitivity and specificity with high accuracy of total antibody testing for SARS-CoV-2 identification. A previous report indicated a high seroconversion of total Ab (93.1%) compared with IgG (82.7%) and IgM (64.7%) alone (11), which is consistent with our results (92.91%). In a recent report on the evaluation of immunoassays (10), it was found that the sensitivity and specificity for the identification of total Ab (90%, 100%) surpassed those of IgA (90%, 93%) and IgG (96%, 65%) alone. These results are consistent with our findings of total Ab (92.91% and 94.30%). Despite the use of a double sandwich ELISA, a more sensitive technique (19) in contrast to our indirect ELISA approach, the sensitivity of the antibody detection assay observed in the first week was 38.3% (11), which was comparatively lower than that reported in the present study (67.86%). The AUC is used to measure the accuracy of a diagnostic test (20), and guidelines indicate that a value of ≥0.90 designates an excellent diagnostic test. Remarkably, our results showed a high AUC for the plant rNP-based ELISA.

The findings from our ELISA based on the detection of total Ab in COVID-19 patients indicated its utility in serosurveys to ascertain the fatality rate in different populations and to identify asymptomatic infections. It can also assist in mapping the kinetics of immune responses by monitoring all the antibodies produced during the complete course of disease progression.

This study, however, is subject to several limitations. First, although our ELISA showed good specificity in healthy subjects, we could not assess the cross-reactivity of N proteins between SARS-CoV-2 and other human coronaviruses. Second, cross-sectional samples were used to determine the dynamics of total Ab, even though the development of the immune response of each patient is highly dynamic. Third, we calculated only the median day of seroconversion in 17 symptomatic patients (17/30, 56%) since we could not obtain the serum from the remaining patients during their early course of infection. Therefore, the median time for the development of total Abs might have been affected. Finally, we evaluated the persistence of total Ab for more than 2 months in 13 symptomatic patients (13/30, 43%); further studies are warranted to better understand the antibody response profile using longitudinal sample collection. Another limitation of our study is that, despite the appropriate conditions, we observed a higher background while utilizing our in-house ELISA with E. coli-expressed rNP. Our future studies will attempt to improve and overcome these limitations.

In conclusion, we found that ELISA based on plant-expressed rNPs provided the most efficient results. This research contributes to our understanding that the detection of total Ab against recombinant nucleoproteins of SARS-CoV-2 by means of ELISA has an important diagnostic value as a serological assay. The findings provide convincing evidence for the standard implementation of plant-based total Ab serological assays in the complementary diagnosis and clinical surveillance of patients with COVID-19.

## MATERIALS AND METHODS

### Patients and source of data.

Between February 2020 and July 2020, patients with confirmed COVID-19 were recruited and grouped according to the presence of COVID-19-associated symptoms. All patients included in this study were not vaccinated against SARS-CoV-2 and remained unvaccinated during the entire follow-up period. These patients were hospitalized at three tertiary care hospitals, as follows: Chosun University Hospital, Yeungnam University Hospital, and Seoul National University Bundang Hospital, South Korea. The total Ab (IgG, IgM, and IgA) responses against SARS-CoV-2 were determined by indirect ELISA based on plant- and E. coli-expressed rNPs. The performance of our in-house total Ab ELISA was further compared with three commercial immunoassays, namely, Standard E COVID-19 total Ab ELISA (SD Biosensor) and EDI novel coronavirus COVID-19 IgG and IgM ELISA. At each time point, the mean optical density at 450 nm (OD_450_) was calculated. The negative controls included 158 serum samples obtained in 2015 and 2017, prior to the COVID-19 pandemic, from healthy individuals at Chosun University Hospital.

For both rNP-based and SD Biosensor ELISAs, 141 serial samples were obtained from 32 recruited patients who were positive for SARS-CoV-2 infection (30 symptomatic [128 sera] and 2 asymptomatic [13 sera]). To compare the performance of in-house total Ab ELISA with the EDI-ELISA, a total of 51 samples from 21 SARS-CoV-2-confirmed patients (19 symptomatic [44 sera] and 2 asymptomatic [7 sera]) were used as positive controls, while 20 samples were used as negative controls.

### Isolation of SARS-CoV-2 and quantitative real-time reverse transcription-PCR (RT-PCR).

To isolate the virus, nasopharyngeal and sputum specimens from each patient were treated with 20× penicillin-streptomycin (Thermo Fisher Scientific, Loughborough, United Kingdom) at 4°C for 1 h and were centrifuged for 20 min at low speed to obtain the viral particle-containing supernatant, which was then inoculated into Vero E6 cells. The cells were suspended in viral cell culture media and incubated at 37°C for 3 to 5 days. The supernatant was then collected for RNA extraction. Viral proliferation was identified after two passages with an interval of 5 days using rRT-PCR with a confirmatory cycle threshold (*C_T_*) value of <20.

RNA from nasopharyngeal and sputum specimens were extracted using the real-prep viral DNA/RNA kit (BioSewoom, South Korea) and fully automated instruments (Bio-seam, South Korea). The extracted RNA was then subjected to rRT-PCR targeting the E gene and RNA-dependent RNA polymerase according to the manufacturer’s instructions (KogeneBiotech, Seoul, South Korea or SD Biosensor, Inc.) (1).

### Recombinant proteins.

The rNPs were produced and synthesized commercially using Nicotiana benthamiana plants (BioApplications Inc., South Korea) and E. coli (Bionics, South Korea). Both proteins had the same 420-amino acid sequence. Protein purity and antigenicity were confirmed by SDS-PAGE and Western blotting, respectively.

### Validation of plant- and E. coli-based rNPs using SDS-PAGE and Western blotting.

To validate the antigenicity and protein purity of plant- and E. coli-based rNPs, we tested a serum sample positive for SARS-CoV-2 infection obtained from a patient in the convalescent phase. For a negative control, pooled negative sera were obtained from the healthy individuals prior to the COVID-19 pandemic. The proteins were separated by 12% sodium dodecyl sulfate polyacrylamide gel electrophoresis (SDS-PAGE) followed by electroblotting of protein bands onto polyvinylidene difluoride (PVDF) membranes (Thermo Fisher Scientific, Germany). The blot was cut into strips and blocked with blocking buffer (phosphate-buffered saline with Tween 20 [PBS-T] containing 5% skimmed milk) for 1 h at room temperature followed by incubation with serum samples as primary antibodies (1:1,000 diluted in blocking buffer) at 4°C overnight. After a wash step in PBS-T for 10 min, the bound antibodies were detected using horseradish peroxidase (HRP)-conjugated secondary antibody goat anti-human IgG (Invitrogen, USA) at a dilution of 1:10,000 in PBS-T for 1 h at room temperature. The immunoprecipitated bands were developed using enhanced chemiluminescence reagents, and the membranes were scanned with an infrared imaging system. The expression and purity of both proteins were identified by SDS-PAGE gel stained with InstantBlue Coomassie protein stain (ab119211).

### Identification of total antibody against rNP of SARS-CoV-2 by ELISA.

Serum antibodies against SARS-CoV-2 were determined using an indirect ELISA. The 96-well ELISA microplates (Thermo Fisher Scientific Korea, Ltd.) were coated overnight at 4°C with 100 μL per well of 2 μg/mL of plant- and E. coli-expressed rNPs. The microplates were washed three times with washing buffer of PBS-T (0.05% Tween 20) and blocked with blocking buffer (PBS-T containing 5% of skim milk) for 2 h at 37°C. After four washes, the specimens were diluted 100-fold with blocking solution and incubated at 37°C for 2 h. The plates were then washed five times with washing buffer. Following this step, HRP-conjugated goat anti-human total Ab (Thermo Fisher Scientific; catalog no. 31418) was diluted in blocking solution (1:40,000) and added at a 100 μL volume per well and incubated at 37°C for 1 h. After an extensive washing step, 50 μL of 3,3′,5,5′-tetramethylbenzidine substrate (TMB; Sigma-Aldrich, USA) was added to each well at room temperature in the dark. After 30 min, the reaction was stopped with 25 μL of 1 M H_2_SO_4_, and the absorbance at 450 nm was measured in each well. The samples were tested in triplicate.

### SD Biosensor Standard E COVID-19 total Ab ELISA.

The assay was performed according to the manufacturer’s instructions (catalog no. E-NCOV-01T; SD Biosensor, Inc. South Korea). The assay was intended to detect total antibodies (IgM/IgA/IgG) to SARS-CoV-2 in human serum by binding to the precoated spike protein on the microplate. The cutoff value was calculated by adding the mean absorbance at 450 nm of the negative control to 0.3.

### EDI novel coronavirus COVID-19 IgG and IgM ELISA.

The assay was performed according to the manufacturer’s protocol (Epitope Diagnostic, Inc., San Diego, CA). The ELISA detects IgG (catalog no. KT-1032)-specific antibodies in human serum by binding to SARS-CoV-2 recombinant full-length nucleocapsid protein coated on the plates. The ELISA that detects IgM-specific antibodies (catalog no. KT-1033) is based on the capture of IgM in human serum and then detects antibodies binding to the SARS-CoV-2 nucleocapsid protein. The cutoff values were calculated by adding the average OD of negative controls to 0.18 (for IgG) or 0.10 (for IgM) and multiplying by 0.9 and 1.1 to obtain the negative and positive results, respectively.

### Data analysis.

Sensitivity was defined as the percentage of patients accurately detected as having COVID-19, as initially diagnosed using rRT-PCR and SARS-CoV-2 culture from respiratory specimens. Specificity was defined as the percentage of patients who were accurately identified as not having SARS-CoV-2 infection. The results of categorical variables were expressed as percentages and counts, whereas continuous variables were expressed as mean and standard deviation or median and interquartile range (IQR). To compare the paired nominal categorical data, an analysis was performed using McNemar’s test. A correlation analysis was performed using Pearson’s correlation coefficient. Statistical significance was set at a *P* value of <0.05. All statistical analyses were performed using MedCalc Software Ltd. (Ostend, Belgium).

### Determination of positive or negative ELISA results by identification of the cutoff value.

To determine the best cutoff value for the total Ab ELISA, we implemented two different calculation methods. (i) The first optimal cutoff value (maximum trade-off between sensitivity and specificity) was identified by generating a receiver operating characteristic (ROC) curve (MedCalc Software Ltd., Ostend, Belgium). To evaluate the accuracy of the test, the area under the concentration-time curve (AUC) was calculated (20). (ii) For in-house total Ab ELISA, the second cutoff value was identified by OD_450_ plus 3-fold standard deviation (mean + 3 SD) by utilizing 158 negative controls. For SD Biosensor total Ab ELISA, 158 negative sera were used, and the cutoff value was obtained according to the manufacturer’s protocol. For EDI-IgG and IgM ELISA, 20 negative controls were utilized, and the cutoff values were calculated using the manufacturer’s method.

### Analysis of ELISA performance using cutoff values and SARS-CoV-2-positive samples.

To analyze the performance of the ELISAs, each of the two obtained cutoff values was evaluated using the defined SARS-CoV-2-positive samples. If the resulting OD value of a patient’s serum sample is greater than the calculated cutoff value, the ELISA result is considered true positive (TP). Conversely, if the OD value is less than or equal to the cutoff value, the assay is considered false negative (FN). This criteria was used to calculate and compare the sensitivity, specificity, and accuracy. The results of these parameters are further analyzed in Table 1.

This study was approved by the institutional review boards (IRB) of Chosun University Hospital (IRB 2020-02-011-003), Seoul National University (IRB B-2008/633-304), and Yeungnam University (IRB 2020-05-080). Written informed consent was obtained from all participants.
