# Supplementary material for: Usefulness of ELISA Using Total Antibody against Plant-Expressed Recombinant Nucleocapsid Protein of SARS-CoV-2
Source: Microbiol Spectr. 2021 Nov 24;9(3):e00672-21. doi: 10.1128/Spectrum.00672-21 (PMC8612163; doi:10.1128/Spectrum.00672-21)
Supplement: SUPPLEMENTAL FILE 1 — Supplemental material. Download SPECTRUM00672-21_Supp_1_seq7.pdf, PDF file, 1.1 MB [file spectrum00672-21_supp_1_seq7.pdf]

**Supplementary Figure 1: Analysis of plant and E. coli expressed SARS-CoV-2 nucleoprotein antigens.**

**(A)** Coomassie blue SDS-PAGE gel and **(B)** Western blotting of plant-based rNP (2µg of concentration) **(C)** Coomassie-blue SDS-PAGE gel and **(D)** Western blotting of *E. coli*-based rNP (2µg of concentration).

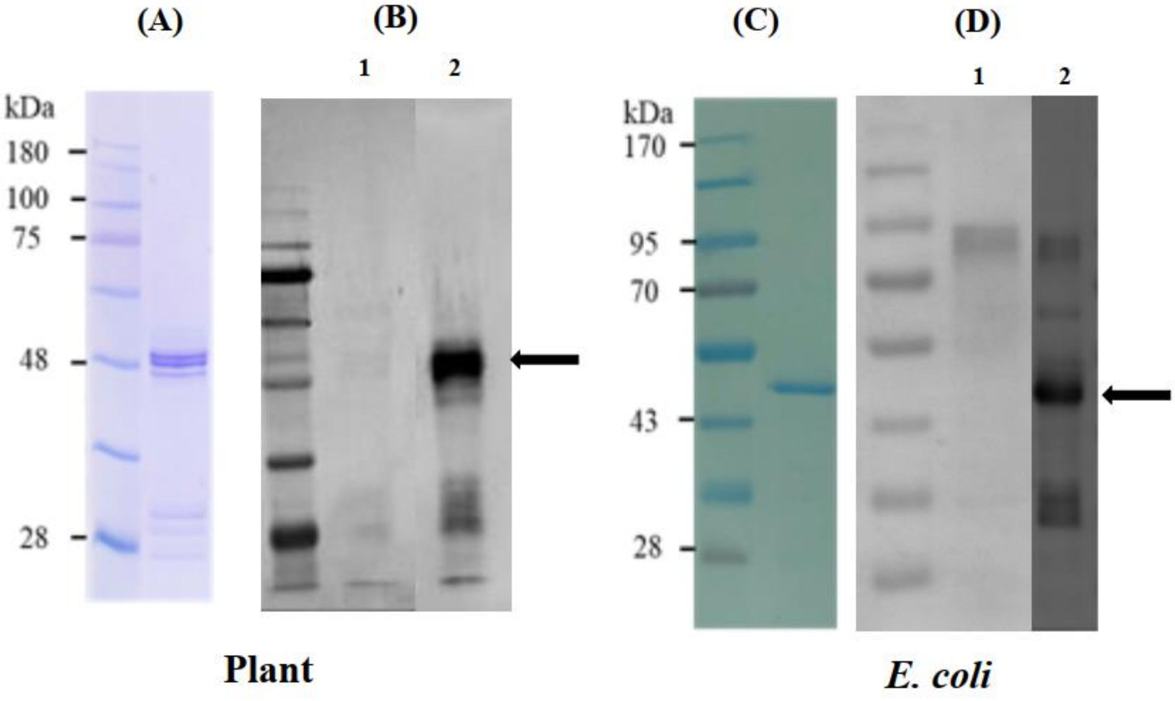


Lines on the left indicated the molecular weight marker (protein ladder) in kDa. The arrows indicates the expected sizes for recombinant N protein derived from plant (48~ kDa) and *E. coli* (~46 kDa). Lanes 1A and 1B were blotted with pooled negative sera, while Lanes 2A and 2B with serum samples positive for SARS-CoV-2 infection obtained from a patient in the convalescent phase.

**Supplementary Figure 2: Optical density at 450 nm (OD450) for the detection of SARS-CoV-2-specific total antibodies, IgG and IgM by weeks after symptom onset.**

**(A)** Plant-based rNP **(B)** *E. coli*-based rNP **(C)** SD Biosensor-Total Ab **(D)** EDI-IgG and **(E)** EDI-IgM ELISA assays. The grey line shows the calculated cut-off values by mean+3SD or range recommended by commercial assay. The red line shows the cut-off values recommended by ROC-curve. Green lines indicate median with interquartile ranges.

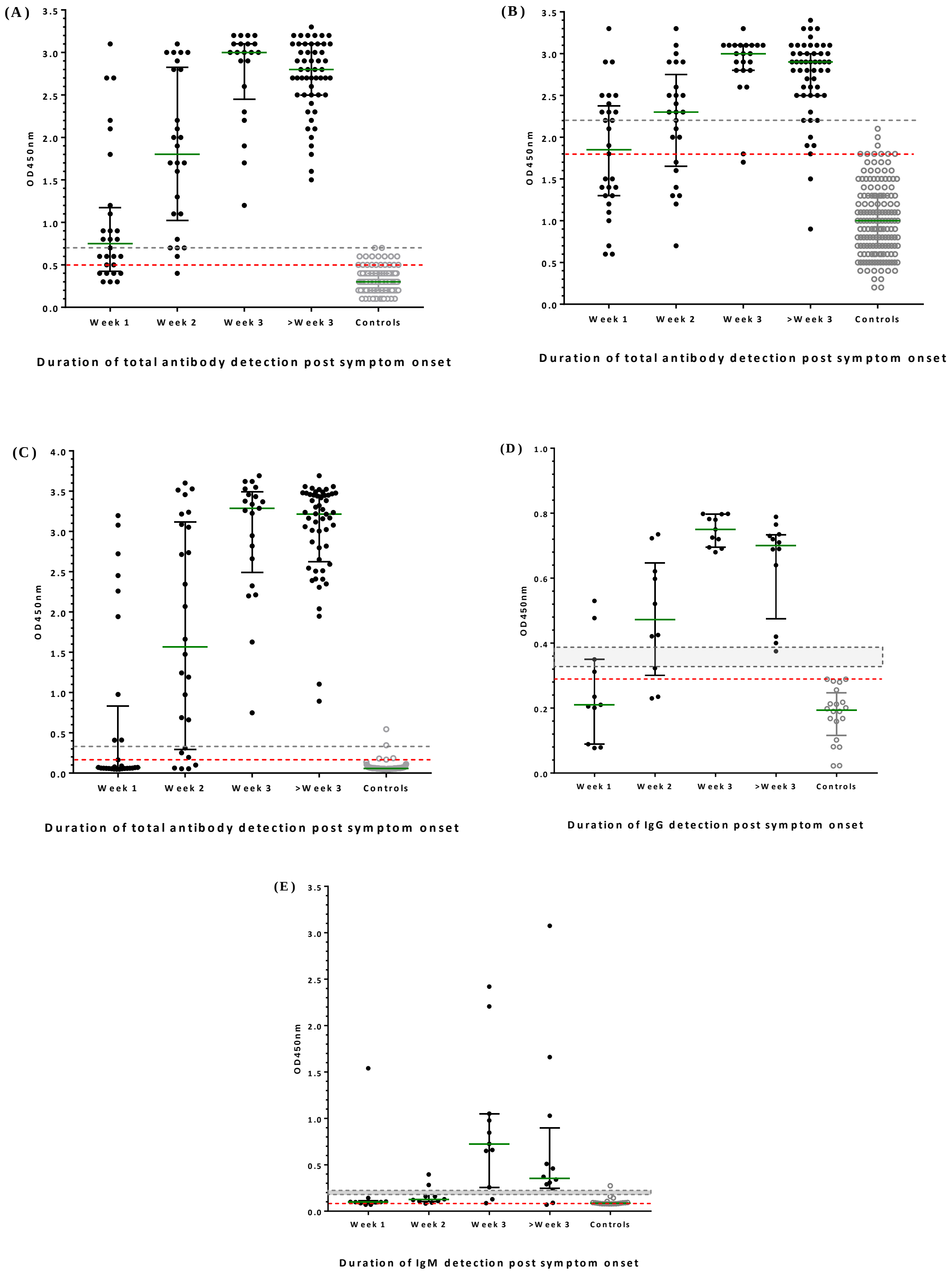
(A)
OD450nm
Week 1
Week 2
Week 3
>Week 3
Controls
Duration of total antibody detection post symptom onset
(B)
OD450nm
Week 1
Week 2
Week 3
>Week 3
Controls
Duration of total antibody detection post symptom onset
(C)
OD450nm
Week 1
Week 2
Week 3
>Week 3
Controls
Duration of total antibody detection post symptom onset
(D)
OD450nm
Week 1
Week 2
Week 3
>Week 3
Controls
Duration of IgG detection post symptom onset
(E)
OD450nm
Week 1
Week 2
Week 3
>Week 3
Controls
Duration of IgM detection post symptom onset

The data set contain 286 samples for rNP Plant-, rNP E. coli-based and SD Biosensor-Total Ab assays; including 128 samples from 30 symptomatic COVID-19 patients and 158 samples from negative controls. For EDI – IgG and IgM ELISA assay 64 samples were analyzed; including 44 samples from 19 symptomatic COVID-19 patients and 20 samples from negative controls.

mean +3SD, mean optical density plus 3 fold of standard deviation

ROC, receiver operating characteristic
